# Optimal Timing of Coronary Artery Bypass Grafting in Haemodynamically Stable Patients after Myocardial Infarction

**DOI:** 10.3390/biomedicines11030979

**Published:** 2023-03-22

**Authors:** Chloé Bernard, Marie Catherine Morgant, Aline Jazayeri, Thomas Perrin, Ghislain Malapert, Saed Jazayeri, Alain Bernard, Olivier Bouchot

**Affiliations:** 1 Department of Cardiac Surgery, Dijon University Hospital, 21000 Dijon, France; 2 Department of Digestive Surgery, Dijon University Hospital, 21000 Dijon, France

**Keywords:** myocardial infarction, surgical revascularization, early, coronary artery bypass graft surgery, prognosis factors

## Abstract

During the acute phase of myocardial infarction, the culprit artery must be revascularized quickly with angioplasty. Surgery then completes the procedure in a second stage. If emergency surgery is performed, the resulting death rate is high; 15–20% of patients are operated on within the first 48 h after the myocardial infarction. The timing of surgical revascularization and the patient’s preoperative state influence the mortality rate. We aimed to evaluate the impact of surgery delay on morbimortality. Between 2007 and 2017, a retrospective monocentric study was conducted including 477 haemodynamically stable patients after myocardial infarction who underwent an urgent coronary bypass. Three groups were described, depending on the timing of the surgery: during the first 4 days (Group 1, *n* = 111, 23%), 5 to 10 days (Group 2, *n* = 242, 51%) and after 11 days (Group 3, *n* = 124, 26%). The overall thirty-day mortality was 7.1% (*n* = 34). The death rate was significantly higher in Group 1 (*n* = 16; 14% vs. *n* = 10; 4.0% vs. *n* = 8; 6%, *p* < 0.01). The mortality risk factors identified were age (OR: 1.08; CI 95%: 1.04–1.12; *p* < 0.001), peripheral arteriopathy (OR: 3.31; CI 95%: 1.16–9.43; *p* = 0.024), preoperative renal failure (OR: 6.39; CI 95%: 2.49–15.6; *p* < 0.001) and preoperative ischemic recurrence (OR: 3.47; CI 95%: 1.59–7.48; *p* < 0.01). Ninety-two patients presented with preoperative ischemic recurrence (19%), with no difference between the groups. The optimal timing for the surgical revascularization of MI seems to be after Day 4 in stable patients. However, timing is not the only factor influencing the death rate: the patient’s health condition and disease severity must be considered in the individual management strategy.

## 1. Introduction

Myocardial infarction (MI) is the main cause of death in developed countries [1]. The ageing of the population combined with the persistence of cardiovascular risk factors is leading to a constant increase in its incidence. However, urgent medical care such as angioplasty, fibrinolysis and antiplatelet drugs has improved survival [2]. In addition to medical treatment, emergency cardiac surgery shows a high rate of morbimortality through the extension and haemorrhage of the infarcted area [3]. Currently, coronary artery bypass grafting (CABG) is a secondary treatment after the early medical and interventional management of MI [4]. In fact, only 5% of MI patients receive CABG as a first treatment [1] and 5% salvage CABG in cases of angioplasty failure [1]. In patients undergoing surgery for MI, the timing of the coronary surgery seems to be a predictive factor of mortality, with a mortality rate of up to 15–20% in patients operated on in the first 48 h after the MI versus 4–5% for those operated on after 48 h [2,4].

Multiple retrospective studies have aimed to assess the timing of surgery as a predictive factor of survival [5,6,7,8]. However, discrepancies in patient characteristics, surgical techniques (off or on pump) and cut-offs for timing prevented strong recommendations [8,9].

The aim of this study was to define the optimal timing for CABG in the early management of haemodynamically stable patients with acute MI. Perioperative events were also assessed.

## 2. Materials and Methods

### 2.1. Ethical Statement

The study was approved by the local ethics committee (EC). The patients’ informed consent was obtained by our clinical research associate.

### 2.2. Study Population

We analysed the data of consecutive patients admitted for MI who were haemodynamically stable at diagnosis with a possible degradation over time and who underwent a CABG, either as a first-line treatment or after angioplasty failure, in our institution from January 2007 to December 2017.

### 2.3. Inclusion and Exclusion Criteria

In this retrospective cohort, MI was defined as the association of thoracic pain, troponin elevation (≥0.10 µg/L) and ECG abnormalities (new, significant ST-T wave changes or a left bundle branch block on a twelve-lead ECG; pathological Q-waves). Cardiogenic shock (Killip 3 and 4) at admission was excluded. Patients with initial multiple organ failure, lactate elevation > 2.0 mmol/L, use of vasopressors or inotropic drugs and/or mechanic ventilation were deemed unstable and excluded. Patients who underwent a combined procedure in addition to CABG (i.e., left ventricular aneurysm, mitral valve repair) were also excluded.

### 2.4. Data Collection

We collected the patients’ clinicopathological features; age, sex, body mass index (BMI kg/m^2^), type 2 diabetes status, systemic hypertension, hyperlipidaemia, smoking, heredity, renal failure, peripheral vascular disease, troponin level (ng/mL), lactate level (mmol/L), coronary history, LVEF (%), EuroSCORE 2, left main disease, number of diseased coronary arteries and ischemic recurrence.

All patients underwent preoperative coronary angiography and antiplatelet administration (i.e., acetylsalicylic acid, clopidogrel or ticagrelor). Each CABG was performed through a median sternotomy incision. CABG techniques and graft use were at the discretion of the treating physicians.

Based on prior reports (literature review), patients were split into three groups according to the “time-to-surgery”, defined as the period between MI and CABG: <4 days, 5–10 days and >11 days (references). The timing of MI was defined as the time between surgery and the first abnormal ECG.

**Figure 1:**
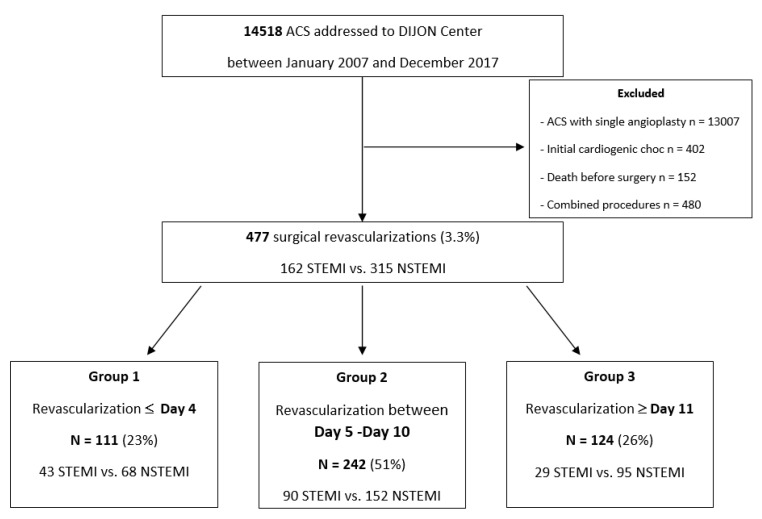
Flow chart.

Group 1 included patients revascularized before Day 4 (*n* = 111), Group 2 included patients vascularized in Days 5–10 (*n* = 242), and Group 3 included patients vascularized after Day 11 (*n* = 124). Patient distribution is described in the flow chart (Figure 1).

We chose these time intervals after a systematic review of the literature and our statistical analysis revealed interesting thresholds. The articles used to conduct the analysis are referred to in the discussion. The period between MI and patient surgery was not arbitrarily defined or randomized. The “time-to-surgery” was allocated for each patient in line with the emergency operative room availability, the delay of the last clopidogrel or ticagrelor administration, haemodynamic degradation after diagnosis and/or ischemic recurrence.

### 2.5. Endpoint Definitions

Surgical mortality was defined as a death status within thirty days after CABG. Preoperative ischemic recurrences were defined as the association of thoracic pain, increased troponin and electric modifications on ECG during the “time-to-surgery” period. Postoperative cardiac or cerebrovascular events were defined as low cardiac output syndrome (hypoperfusion signs and alteration of LVEF ± hyperlactatemia), cardiogenic shock, cardiac arrest, surgical exploration for mediastinal bleeding and strokes occurring after CABG.

### 2.6. Statistics

Continuous variables were expressed as medians (interquartile ranges or means (standard deviation), if appropriate) and categorical variables as percentages. In a univariate analysis, continuous variables were compared using Student’s *t*-test. Categorical variables were compared with the Chi2 test. Preoperative variables with a *p* < 0.2 in the univariate analysis were deemed significant and included in the multivariate analysis. For the multivariate analysis, a logistic regression model was used. The adequacy of the model was tested using Hosmer–Lemeshow. The performance of the test was then assessed using the ROC curve. Statistical analysis was performed with STATA version 14 (StataCorp, College Station, TX, USA).

## 3. Results

### 3.1. Study Population

#### 3.1.1. Preoperative State

During this period, 14,518 patients were admitted for acute coronary syndrome (ACS) in our centre. Among them, 477 (3.3%) underwent coronary bypass. In our population, 162 patients (34%) presented with a STEMI and 315 (66%) with a NSTEMI. Baseline patient characteristics are summarized in Table 1. The three groups were similar except for hyperlipidaemia, which was less represented in Group 2, with 72% vs. 60% vs. 71% (*p* = 0.02). The mean patient age was 67 ± 12 years, and 23.6% of patients were women. The most-represented cardiovascular risk factors were hyperlipidaemia (68%), systemic hypertension (68%) and smoking (56%). Thirty-three patients had preoperative kidney failure (7%), defined as a creatinine level > 200 µmol/L. Every patient benefited from preoperative coronary angiography and antiplatelet administration (aspirin *n* = 420, clopidogrel *n* = 205, ticagrelor *n* = 90).

There were significantly fewer STEMIs in Group 3: 23% vs. 39% (Group 1) and 37% (Group 2) (*p* = 0.015). The distribution of left main disease was homogeneous between the groups and involved 48% of the overall population. The number of diseased coronary arteries was comparable between the three groups. Most patients had conserved left ventricular function and the mean left ventricular ejection fraction (LVEF) was 53 ± 11.4%. A reduced LVEF (≤40%) was reported in 17% of the overall population, with 21% in Group 1 (*n* = 23), 18% in Group 2 (*n* = 44) and 12% in Group 3 (*n* = 15). Mean lactate (*p* = 0.54) and mean troponin maximal levels (*p* = 0.32) were comparable between the groups, with 1.8 mmol/L (0.48–3.8 ± 2.26) and 25 ng/mL (0.02–530 ± 54) respectively.

No patient needed intubation, inotropic medication or vasopressors, initially.

Immediately after the diagnostic angiography, 41 patients (8.6%) had a percutaneous coronary intervention with no effective result, and the remaining 436 patients were ineligible because of unfriendly anatomy or three-vessel disease (Table 2). Among those 41 patients, 32 had a STEMI. There were differences in cardiologic preoperative management. Group 1 required more angioplasty procedures (*n* = 17 (15%) vs. *n* = 10 (4%) vs. *n* = 14 (11%), *p* < 0.01). Concerning pharmacological management, there were fewer preoperative antiplatelet treatments, including aspirin, in Group 2 (*n* = 207 (85%) vs. *n* = 104 (94%) vs. *n* = 116 (93%), *p* = 0.01). The last revascularized group (Group 3) received significantly more aspirin treatment (*n* = 116 (94%) vs. *n* = 99 (89%) vs. *n* = 205 (85%), *p* = 0.01).

Twenty percent of the overall population (*n* = 92) had an ischemic recurrence before revascularization surgery, 25 in Group 1 (22%), 37 in Group 2 (16%) and 30 in Group 3 (24%), with no significant difference between the groups (*p* = 0.2) (Table 3). Among those recurrences, 34 ultimately required an intra-aortic balloon pump (37%) to hold on until surgery. Over time, recurrences were complicated by acute pulmonary oedema (*n* = 11, 12%), cardiorespiratory arrest (*n* = 4, 4.3%) or cardiogenic shock (*n* = 4, 4.3%). In those cases, patients became unstable and required emergency revascularization surgery in poorer conditions.

The mean revascularization delay was 10.5 ± 13.2 days.

#### 3.1.2. Intraoperative Data

Each operation involved a median sternotomy incision. Cardiopulmonary bypass use depended on the patient’s cardiac function and the individual surgeon’s practice. If LVEF was less than 40%, we usually performed a CPBP. The CPBP was performed in a central fashion: an artery canula in the ascending aorta and a veinous canula in the right atrium. Cardioplegia was usually administered with Custodiol (EUSA Pharma, Limonest, France) under middle hypothermia (32 °C).

The type of graft (internal thoracic artery or saphenous vein) depended on the surgeon and the patient’s age. Vein grafts were used on the right coronary artery in patients older than 65 years.

Most CABG procedures were performed on-pump (80%), *n* = 380 vs. *n* = 97 using cardioplegic arrest, with no difference between the groups. There were two on-pump, beating-heart coronary artery bypass grafts. The mean bypass and cross-clamping times were similar in the three groups: 66 ± 31 vs. 70 ± 44 vs. 71 ± 57 min, *p* = 0.8 and 44 ± 33 vs. 51 ± 27 vs. 50 ± 31 min, *p* = 0.1, respectively. Patients received a mean of three distal anastomoses. At least one internal thoracic artery was used in 97% of patients in all groups. Both were used in 98% of cases of bitroncular lesions, and saphenous veins were added in 77% of cases of tritroncular lesions. No radial artery was used. Complete revascularization was achieved at comparable rates among the groups (84.4% vs. 91.6% vs. 87%, *p* = 0.67).

### 3.2. Mortality

The overall thirty-day mortality, adjusted for various preoperative factors, was 7% of the total population.

The mortality rate was significantly higher in Group 1 (*n* = 16 (14%)) vs. Group 2 (*n* = 10 (4%)) vs. Group 3 (*n* = 8 (6%)), *p* < 0.01 (Table 4). Death aetiologies were significantly different among the groups. In Group 3, the main cause of death was multiorgan failure (*n* = 3 (19%) vs. *n* = 1 (10%), vs. *n* = 5 (62%), *p* = 0.023), whereas cardiac causes (cardiorespiratory arrest or cardiogenic shock) were mostly represented in Group 1 (*n* = 7 (44%) vs. *n* = 2 (20%) vs. *n* = 2 (25%), *p* < 0.01).

Concerning the overall thirty-day mortality, cardiac complications were the first cause of death (*n* = 11, 32%), followed by multiorgan failure (*n* = 9, 26.5%) and other causes, such as stroke (*n* = 1, 2.7%) and mesenteric ischemia (*n* = 2, 5.8%). One death was due to an early CABG thrombosis on Day 1.

### 3.3. In-Hospital Outcomes and Poor Prognosis Factors

#### 3.3.1. Risk Factors

Some highlighted risk factors were linked to patient physiology: age (OD: 1.08; CI 95%: 1.04–1.12; *p* < 0.001), preoperative kidney failure (OD: 6.39; CI 95%: 2.49–15.6; *p* < 0.001), peripheral vascular disease (OD: 3.31; CI 95%: 1.16–9.43; *p* = 0.024) and preoperative ischemic recurrence (OD: 3.47; CI 95%: 1.59–7.48; *p* < 0.01) (Table 5).

Preoperative LVEF, type of MI and left main disease were not associated with a higher risk of postoperative death.

Pre- and intraoperative transfusion (*p* = 0.353), cross-clamping time (*p* = 0.353) or the number of anastomoses (*p* = 0.503) were not significantly associated with thirty-day mortality.

Ischemic recurrence was significantly associated with mortality (OD: 3.47; CI 95%: 1.59–7.48; *p* < 0.01).

#### 3.3.2. Postoperative Outcomes

Postoperative complications had a homogeneous distribution between the groups except for the septic shock rate, which was significantly higher in Group 1 (*n* = 4, 3.6% vs. *n* = 0.0% vs. *n* = 4, 0.8%, *p* = 0.042) (Table 6). Eighteen patients underwent immediate redo surgery for bleeding (*n* = 6 (5.4%) in Group 1, *n* = 9 (3.7%) in Group 2 and *n* = 3 (2.4%) in Group 3, *p* = 0.54). Six patients (1.2%) presented with early ischemic symptom recurrence after surgery (< 30 days) and 26 (5.2%) with later ischemic symptom recurrence (>30 days). Eleven patients experienced cardiac failure (2.3%), seven stroke (1.4%), fifteen late cardiogenic shock (3.1%) and two acute pulmonary oedema (0.4%), with no significant difference between the groups. Finally, 18 patients (3.7%) needed a wound reoperation for infection and two went into septic shock. Two other septic shocks were secondary to pulmonary infection.

### 3.4. Follow-Up

The mean follow-up was 60 ± 45 months. Sixty-five patients died during the follow-up (14.6%), with one endocarditis and three fatal digestive bleedings.

The complete follow-up was 95.3%.

## 4. Discussion

Surgical revascularization timing after MI is still a subject of debate. Since 2018, according to ESC and EACTS Guidelines, Heart Teams must provide a balanced, multidisciplinary decision-making process for every case of myocardial infarction [10] to offer each patient the most appropriate treatment for their overall health profile and the severity of their cardiac disease. Klempfnet et al. showed a reduction in the number of patients referred for emergency coronary bypass in the last ten years, probably in response to the procedure’s known high mortality (6.7% in 2000 vs. 1.7% in 2010, *p* < 0.001) and the efficacy of angioplasty. Emergency bypass mortality did not decrease for 20 years (14.3% vs. 10%, *p* = 0.15), unlike the mortality of elective surgery [10].

The literature on this subject is heterogeneous, and most studies show no significant difference between early and delayed revascularization surgery on thirty-day mortality [5].

The goals of myocardial revascularization are: preserving the remaining myocardial function, preventing further functional deterioration and ischemic recurrence, and recruiting hibernating myocardium to improve ventricular function. These arguments prompt some authors to recommend revascularization during the early period.

Our study suggests that bypass grafting should not be performed before four days after MI. Grothusen et al. proposed an optimal timing of 48 h for NSTEMI, with no significant difference in mortality, ischemic recurrence, cardiac failure or cardiogenic shock between their groups [6]. For Piroze et al., this period can be brought down to 24 h for STEMI [11]. Assman et al. reported results similar to ours and advocated delaying surgery for three days for stable patients (STEMI and NSTEMI) [12]. Parikh et al. showed in NSTEMI that high-cardiovascular-risk patients revascularized after 48 h had the same mortality as low-risk patients revascularized before 48 h [5]. A meta-analysis published in 2014 described a “U”-shaped curve in the postoperative mortality of emergency revascularized patients [3]. In this study, patients revascularized between Days 3 and 5 had lower mortality than the others. The authors explained that during the acute period, there is a major inflammatory state that increases mortality after emergency bypass, justifying its postponement. Maganti et al. insisted that early revascularization surgery is unsafe for patients with a high cardiovascular risk [13]. This trend was confirmed by Klempfner et al. in 2016 [9]. All these results seem to show that low-risk patients could benefit from early surgery, leaving a short period of 48 h, whereas delayed surgery should be preferred in high-risk patients. This additional time should allow for partial patient recovery and the medical optimization of their general state and comorbidities. Some teams differentiated between STEMI and NSTEMI, which was not the case in our study. We decided to conduct a comprehensive analysis with a larger workforce. Group 3 included more NSTEMI, probably because those patients are considered less severe than STEMI in our institution and can wait for surgery.

In our study, revascularization timing did not only depend on the patient’s state but sometimes on previous medical treatment: anticoagulants and antiplatelet agents (ticagrelor) meant delaying surgery. Unfortunately, we also depended on surgical planning.

Many studies have confirmed the increased bleeding risk in patients who receive clopidogrel five to seven days before surgery, yet its administration before coronary angiography is associated with a significant decrease in ischemic recurrence thirty days after STEMI [14]. The benefit/risk ratio must be individually evaluated.

A relevant fact in our study is that preoperative ischemic recurrence is a risk factor for thirty-day mortality. We did not find any other recent studies describing this parameter as a risk factor. We defined our “time-to-surgery” period in such a way as to observe this ischemic recurrence and its impact on mortality. According to Grieshaber et al., a significant proportion of patients (12% in their study) who were assigned to delayed surgery developed low cardiac output syndrome (LCOS) during the waiting period [15]. These risks of deadly complications justify an attempt to reduce this waiting period. From Group 3, it seems that the more we delay surgery (over Day 11), the more likely patients will present with ischemic recurrence.

The mortality of this revascularization surgery is linked to various preoperative factors also highlighted in the literature. Increased mortality in patients operated on soon (<48 h) after MI seems to be linked to multiple risk factors such as age > 65 years old [6,7,8], poor LVEF [6,9,13], acute renal failure [9] or anaemia [16]. Left ventricle failure is one of the major complications of ischemia that must be strongly considered before STEMI revascularization surgery. Other risk factors, including biology with elevated troponin levels [17] and hyperlactatemia [9], reflect the seriousness of ACS.

The majority of teams perform on-pump CABG (94%) [12]. However, in theory, off-pump surgery appears to be the best strategy because it avoids “over-ischemia” thanks to the use of shunts and the absence of cardioplegia [18]. Moreover, it reduces ischemia-reperfusion injury and decreases oxidative and inflammatory stress. In practice, off-pump CABG has never shown superiority over on-pump in MI management. For some authors, such as Fattouch et al., beating-heart surgery must be limited to early revascularization (<48 h) [19]. In our set of patients, we did not demonstrate any difference in morbidity-mortality between the strategies, irrespective of revascularization timing.

### Limitations

Given the retrospective nature of our study, there are some missing data. We did not know the stopping day for antiplatelet or anticoagulant treatment, which would be interesting to interpret the results of bleeding complications and mortality-morbidity. We regret that the blood loss during surgery is unknown. The major limitation was the lack of comparability between the three groups concerning the preoperative treatment and the secondary instability and ischemic recurrence, which are probably linked to the duration of the waiting period. The best strategy would likely be to conduct a randomized controlled study; however, in this situation, that may raise ethical issues. Moreover, this study does not provide any clarifications about the management of instable patients, which remains unclear.

## 5. Conclusions

Our study suggests that haemodynamically stable patients after MI should benefit from surgical revascularization at least four days after their acute coronary syndrome. Classic mortality risk factors have been identified, such as age, preoperative kidney failure and peripheral vascular disease. Preoperative ischemic recurrence is a new risk factor of mortality identified in this study. Avoiding ischemic recurrence is likely key to the preoperative management of those patients. Therefore, timing management, overall health state and an effort to limit ischemic recurrence must be considered by Heart Teams in order to provide the best decision.

## Figures and Tables

**Table 1:** Preoperative patient characteristics.

Variables	Group 1 *n* = 111	Group 2 *n* = 242	Group 3 *n* = 124	*p*-Value
Age (years)	65 ± 12	67 ± 12	67 ± 12	0.053
Female sex, *n* (%)	29 (26)	53 (22)	29 (23)	0.57
Comorbid disease				
Body mass index >30, *n* (%)	25 (22)	50 (21)	28 (22)	0.80
Systemic hypertension, *n* (%)	76 (68)	162 (67)	86 (69)	0.89
Hyperlipidaemia, *n* (%)	80 (72)	146 (60)	88 (71)	0.02
Diabetes, *n* (%)	27 (24)	57 (23)	41 (33)	0.13
Smoking, *n* (%)	61 (55)	137 (57)	198 (56)	0.74
Peripheral vascular disease, *n* (%)	12 (11)	20 (8)	11 (9)	0.50
Heredity, *n* (%)	31 (28)	68 (28)	99 (28)	0.93
Renal failure, *n* (%)	5 (4)	20 (8)	8 (6)	0.42
Mean maximal troponin level (ng/mL)	24.8 ± 54	22.9 ± 55	24.6 ± 53	0.32
Mean lactate level (mmol/L)	1.8 ± 1.8	1.5 ± 1.9	1.9 ± 2.2	0.54
Coronary history, *n* (%)	18 (16)	36 (15)	27 (22)	0.29
LVEF (%)	52.8 ± 11.8	52.9 ± 11.3	54.8 ± 11.2	0.3
EuroSCORE II (%)	3.25 ± 1.8	3.0 ± 1.8	3.1 ± 2.7	0.8
STEMI, *n* (%)	43 (39)	90 (37)	29 (23)	0.015
Left main disease, *n* (%)	52 (47)	123 (51)	55 (44)	0.48
Number of diseased coronary arteries				
One, *n* (%)	3 (3)	5 (2)	3 (2)	0.47
Two, *n* (%)	24 (21)	58 (24)	23 (19)	0.52
Three, *n* (%)	84 (76)	179 (74)	98 (79)	0.38
Ischemic recurrence, *n* (%)	25 (22)	37 (15)	30 (24)	0.20

**Table 2:** Preoperative management.

Variable	Group 1 *n* = 111	Group 2 *n* = 242	Group 3 *n* = 124	*p*-Value
Angioplasty failure, *n* (%)	17 (15)	10 (4)	14 (11)	<0.01
Overall antiplatelet use, *n* (%)	104 (94)	207 (85)	116 (93)	0.01
Aspirin, *n* (%)	99 (89)	205 (85)	116 (94)	0.01
Plavix, *n* (%)	55 (50)	103 (43)	47 (38)	0.15
Other antiplatelet agent, *n* (%)	23 (21)	49 (20)	18 (15)	0.35
VKA, *n* (%)	3 (3)	6 (2)	2 (2)	0.85
Fibrinolysis, *n* (%)	12 (11)	26 (11)	10 (8)	0.69

**Table 3:** Events during the waiting period.

Events	Group 1 *n* = 111	Group 2 *n* = 242	Group 3 *n* = 124	*p*-Value
None, *n* (%)	86 (77)	206 (85)	94 (76)	0.13
Ischemic recurrency, *n* (%)	18 (16)	28 (12)	25 (20)	0.13
Cardiorespiratory arrest, *n* (%)	0 (0)	3 (1)	1 (0.8)	
IABP, *n* (%)	53 (48)	42 (17)	6 (5)	<0.001
Cardiogenic shock, *n* (%)	2 (2)	1 (0.4)	1 (1)	
Intra-aortic balloon pump after ischemic recurrence, *n* (%)	16 (14)	16 (6)	2 (2)	<0.001

**Table 4:** Aetiologies of thirty-day mortality.

Outcomes	Group 1 *n* = 111	Group 2 *n* = 242	Group 3 *n* = 124	Total	*p*-Value
Thirty-day mortality, *n* (%)	16 (14)	10 (4)	8 (6)	34 (7)	<0.01
Causes of death					
Multiorgan failure, *n* (%)	3	1	5	9	0.023
Cardiorespiratory arrest or cardiogenic shock, *n* (%)	7	2	2	11	<0.01
Stroke, *n* (%)	0	1	0	1	1
Mesenteric ischemia, *n* (%)	0	2	0	2	0.74
Others, *n* (%)	6	4	1	11	0.054

**Table 5:** Mortality risk factor analysis.

Variables	Odds Ratio	95% Confidence Interval	*p* > |z|
Age	1.08	(1.04; 1.12)	<0.001
Renal failure, *n* (%)	6.39	(2.49; 15.6)	<0.001
Peripheral vascular disease, *n* (%)	3.31	(1.16; 9.43)	0.024
LVEF (%)	0.975	(0.945; 1.01)	0.12
STEMI, *n* (%)	0.701	(0.306; 1.50)	0.38
Left main disease, *n* (%)	1.02	(0.495; 2.07)	0.96
Aspirin, *n* (%)	0.637	(0.260; 1.81)	0.35
Angioplasty failure, *n* (%)	0.227	(0.0968; 0.507)	<0.001
Preoperative intra-aortic balloon pump, *n* (%)	3.26	(1.55; 6.74)	<0.01
Ischemic recurrence, *n* (%)	3.47	(1.59; 7.48)	<0.01
Revascularization timing	3.69	(1.77; 7.57)	<0.001
Postoperative bleeding	0.276	(0.116; 0.630)	<0.01

**Table 6:** Postoperative outcomes.

Postoperative Outcomes	Group 1 *n* = 111	Group 2 *n* = 242	Group 3 *n* = 124	Total	*p*-Value
Redo for bleeding, *n* (%)	6 (5.4)	9 (3.7)	3 (2.4)	18 (3.7)	0.54
Early ischemic recurrence <D3, *n* (%)	0 (0)	3 (1.2)	3 (2.4)	6 (1.2)	0.6
Delayed ischemic recurrence, *n* (%)	8 (7)	11 (4.5)	7 (5.6)	26 (5.2)	0.59
Cardiac failure, *n* (%)	3 (2.7)	6 (2.5)	2 (1.6)	11 (2.3)	0.85
Stroke, *n* (%)	3 (2.7)	4 (1.6)	0 (0)	7 (1.4)	0.52
Cardiorespiratory arrest or cardiogenic shock, *n* (%)	5 (4.5)	7 (2.8)	3 (2.4)	15 (3.1)	0.62
Acute pulmonary oedema, *n* (%)	2 (1.8)	0 (0)	0 (0)	2 (0.4)	0.054
Wound surgery for infection, *n* (%)	4 (3.6)	10 (4.1)	4 (3.2)	18 (3.7)	0.92
Septic shock, *n* (%)	4 (3.6)	0 (0)	0 (0)	4 (0.8)	0.042

## Data Availability

The data presented in this study are available on request from the corresponding author.
